# HIV effects on age-associated neurocognitive dysfunction: premature cognitive aging or neurodegenerative disease?

**DOI:** 10.1186/s13195-015-0123-4

**Published:** 2015-04-06

**Authors:** Ronald A Cohen, Talia R Seider, Bradford Navia

**Affiliations:** Departments of Neurology, Cognitive Aging and Memory Program, Institute on Aging, Psychiatry, and Aging and Geriatric Research, University of Florida, 2004 Mowry Road, Gainesville, FL 32610 USA; Department of Clinical and Health Psychology, University of Florida, 1225 Center Drive, Room 3151, Gainesville, FL 32611 USA; Department of Public Health and Community Medicine, Tufts University School of Medicine, 150 Harrison Avenue, Boston, MA 02111 USA

## Abstract

Marked improvements in survival and health outcome for people infected with HIV have occurred since the advent of combination antiretroviral therapy over a decade ago. Yet HIV-associated neurocognitive disorders continue to occur with an alarming prevalence. This may reflect the fact that infected people are now living longer with chronic infection. There is mounting evidence that HIV exacerbates age-associated cognitive decline. Many middle-aged HIV-infected people are experiencing cognitive decline similar that to that found among much older adults. An increased prevalence of vascular and metabolic comorbidities has also been observed and is greatest among older adults with HIV. Premature age-associated neurocognitive decline appears to be related to structural and functional brain changes on neuroimaging, and of particular concern is the fact that pathology indicative of neurodegenerative disease has been shown to occur in the brains of HIV-infected people. Yet notable differences also exist between the clinical presentation and brain disturbances occurring with HIV and those occurring in neurodegenerative conditions such as Alzheimer’s disease. HIV interacts with the aging brain to affect neurological structure and function. However, whether this interaction directly affects neurodegenerative processes, accelerates normal cognitive aging, or contributes to a worsening of other comorbidities that affect the brain in older adults remains an open question. Evidence for and against each of these possibilities is reviewed.

## Introduction

HIV continues to be a major public health problem [1]. During the early years of the HIV epidemic, the cognitive and functional consequences of HIV were devastating for patients and their families [2]. HIV-associated encephalopathy and dementia were among the most common diagnoses in people with AIDS at the time of death [3,4].

The proportion of HIV-infected people who are older than 45 years of age is approaching 50% in the US and other developed nations and this is due in large part to the effectiveness of antiretroviral therapies [5]. HIV-infected adults over age 55 comprise the fastest-growing age group in the HIV-positive population [6], and advanced age at the time of seroconversion increases the risk for neurocognitive impairment [7]. These epidemiological trends point to the potential significance of the effects of HIV on the aging brain.

## Neurocognitive manifestations

Prior to the availability of antiretroviral drugs, dementia occurred in over 20% of HIV-infected people [4]. The term AIDS dementia complex (ADC) was coined as a diagnosis of severe decline secondary to HIV, typically involving areas of cognitive, motor, and behavioral function [3]. Patients with severe ADC usually experienced the greatest impairments in attention, working memory, and executive functions, along with fine motor and information processing speed [8,9]. Primary amnestic disturbances did not typically occur, and language, semantics, comprehension, visual-spatial processing, and other sensory and perceptual functions were usually preserved. Although brain disturbances due to opportunistic infections acquired during periods of severe immunosuppression were common [10], ADC was shown to be directly related to HIV infection, predominantly involving macrophages, in the absence of opportunistic infection [11].

In recent years, diagnostic classification of HIV-associated neurocognitive disorder (HAND) was developed as an alternative to ADC staging. HAND encompasses a range of cognitive impairment from mild cognitive difficulties with no functional impairment (asymptomatic neurocognitive impairment, or ANI) to cognitive difficulties with mild functional impairment (mild neurocognitive disorder, or MND) to dementia with significant functional impairment (HIV-associated dementia, or HAD). The advent of combination antiretroviral therapy (cART) in the late 1990s led to reductions in HIV-associated mortality and morbidity [5] and a precipitous decline in incidence of dementia [12]. Overall, cART use led to improved cognitive functioning [13] and reduced neurological damage [14]. Yet HAND continues to occur in 30% to 50% of infected people [8,15-17]. In the cART era, as before, cognitive and motor slowing are major elements of HAND, along with impairments of attention, working memory, and executive functioning [17]. Learning efficiency is reduced, along with memory retrieval, although primary amnestic disturbances are still rare. Though less severe than dementia, ANI and MND affect occupational and psychosocial functioning, quality of life, and health outcomes [9,18,19].

### Age-associated cognitive decline in HIV

For several reasons, the effect of HIV on the aging brain has become the subject of much greater concern over the past decade. First, HIV has become a chronic illness, with infected people now having nearly normal life expectancy [20]. Second, there has been a significant increase in the number of older adults living with HIV. Third, although cART has been very effective in reducing viral replication and AIDS and restoring immunological function, HAND remains prevalent. Finally, there is mounting evidence that HIV and aging may interact to adversely affect the brain and neurocognitive functions.

**Figure 1:**
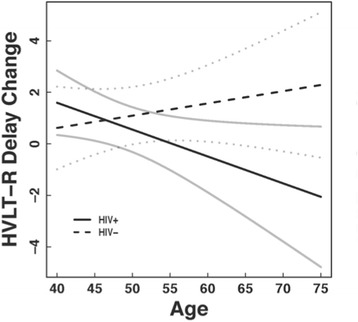
**Change in delayed recall performance on the Hopkins Verbal Learning Test Revised (HVLT-R) as a function of age for HIV** ^**+**^ **and HIV** ^**−**^ **groups.** The HIV^+^ group showed performance declines with increasing age, whereas the HIV^−^ group had either stable or slight improvements in recall over 1 year, showing a clear interaction between age and HIV effects on verbal recall. Results are displayed as means with 95% confidence bands. Change is defined as the difference between baseline and follow-up scores (12 months - 0 months).

Advanced age is among demographic factors associated with reduced neurocognitive performance and susceptibility to HAND in HIV-infected people [21-24], as greater neurocognitive impairment exists among older HIV-infected adults relative to normative data and compared with younger infected individuals. Although greater cognitive and neurological deficits in older people with HIV may result from independent additive effects of the pathophysiological mechanisms of aging and HIV [25,26], longitudinal studies show significant interaction effects of HIV and age [27,28], suggesting that the mechanisms are synergistic. For example, Seider and colleagues [27] showed that older people with HIV showed significant memory decline in 1 year, but no decline was seen in younger people with HIV or in seronegative controls regardless of age (Figure 1). These data indicate that HIV is associated with accelerated cognitive aging such that people with HIV in their 50s and 60s are functioning cognitively more like people typically do in their 70s and 80s. It is interesting to note that problems with learning and memory are reported to a greater extent in the cART era [15,16,29-31], indicating a change in the typical presentation of HAND in older adults with HIV. These changes as well as neuroimaging and neuropathological findings described below raise the question of whether typical age-related neurodegenerative diseases, particularly Alzheimer’s disease (AD), are affecting the development of HAND. In the sections that follow, evidence for and against the idea that AD is contributing to HAND will be discussed, as will research findings that address some of the mechanisms underlying HAND and how they may escalate as infected people reach advanced age.

## Neuroimaging

HIV-associated cortical and subcortical volume reductions, white matter changes, metabolite abnormalities, and regional glucose metabolism that vary relative to HIV clinical factors (for example, viral load, nadir CD4), and HAND severity are evident on magnetic resonance imaging (MRI), magnetic resonance spectroscopy (MRS), and positron emission tomography (PET) [32-34]. Although neuroimaging abnormalities are usually most significant in cases of opportunistic brain infection, HIV also has direct and indirect effects on brain structure and function. Historically, research focused on the basal ganglia and cerebral white matter, regions considered to be particularly vulnerable to HIV [35]. Yet when compared with seronegative controls, people with HIV also show reduced grey matter and cortical thinning [32-34,36], especially in frontal and temporal regions [37]. A study of asymptomatic individuals with HIV revealed decreased frontal grey matter volumes in the absence of other brain changes [38], suggesting that cortical atrophy begins in frontal areas. White matter hyperintensities (WMHs) on MRI reflect white matter damage, and studies show WMHs occurring at younger ages in people with HIV than they do in adults aging without HIV [39,40]. Case-controlled diffusion tensor imaging (DTI) studies show that HIV is associated with lower white matter integrity globally [41] and in specific areas such as the corpus callosum, internal capsules, and the frontal and parietal lobes [42,43]. There is evidence that the earliest HIV-associated white matter effects occur in the frontal lobes [44], with more widespread damage occurring as the disease increases in severity [43,45]. MRS studies show increased myoinositol (MI), choline (Cho), and total creatine (Cr) in brain disorders with chronic inflammation and glial activation, including HIV [32,34,46,47]. N-acetylaspartate (NAA), a marker of neuronal integrity that decreases in response to neuronal damage, has been shown to be lower in people with HIV compared with age-matched controls, especially in the basal ganglia and frontal white matter [46,48]. Although NAA associations with plasma HIV-RNA show that neuronal injury is related to current viral replication, neuronal injury is also found in virologically suppressed patients and may be attributed to the effects of chronic immune activation and inflammation. PET studies have shown that glucose hypometabolism in the frontal cortex, suggesting deficient functioning, and basal ganglia hypermetabolism occur in HIV [49]. Increased basal ganglia metabolism may seem counterintuitive, although HIV-infected astrocytes require increased glucose to proliferate [50], and the basal ganglia are known to be particularly vulnerable to HIV [35].

The neuroimaging abnormalities that have been observed historically among HIV-infected people are similar to those observed among older adults without HIV. As people reach advanced age, cortical and subcortical volumes gradually decrease [51]. Additionally, older age in healthy cohorts has consistently been found to be one of the most important independent predictors of greater WMH volume [52]. WMHs in frontal and parietal regions have been especially associated with older age and greater cognitive dysfunction [53], and longitudinal studies show greater age-related volumetric decline in anterior versus posterior white matter regions [54]. Declines in DTI measures of white matter integrity also occur with increased age [55], with anterior regions showing the greatest changes [56]. Furthermore, MRS research indicates that there is an age-related decline in NAA and increases in Cho and Cr [57]. Finally, PET research shows age-related declines in glucose metabolism, beginning with frontal lobe changes [58].

cART-era research shows that the neuropathology of HIV appears to be changing in that it now involves cortical as well as subcortical structures [33]. This signifies that HIV may progress to involve processes that bear a greater resemblance to age-related neurodegenerative diseases, such as AD, of which cortical atrophy and ventricular enlargement are hallmarks [59]. As in HIV, WMHs have been shown to occur in frontal and parietal lobes in AD, and the degree of WMH in parietal lobes and posterior periventricular areas corresponds with level of cognitive impairment [60]. AD is also associated with widespread DTI abnormalities [61]. However, cortical changes in AD cases are typically more pronounced than in cases of HIV, and hippocampal atrophy occurs early and ubiquitously in AD whereas the hippocampus is not as vulnerable in HIV [62,63]. Also, unlike in HIV, in AD the largest DTI effects are in hippocampal areas. A recent review of MRS abnormalities in AD showed NAA decreases and MI increases similar to HIV. In AD, decreased NAA is generally found in all major lobes of the brain as well as the medial temporal lobe and the posterior cingulate gyrus [64]. MI increases are also common, and changes in NAA and MI are associated with level of AD neuropathology. PET research in AD shows parietal, temporal, and posterior cingulate glucose metabolism decreases that predict cortical volume loss [65], with decreases in the frontal cortex as the disease progresses [66], whereas in HIV frontal hypometabolism is seen early on in the disease.

## Neuropathology and pathophysiology

HIV enters the brain soon after infection, and the brain continues to be a reservoir for HIV even among patients who receive cART [67]. The absence of circulating HIV-RNA in the blood and cerebrospinal fluid (CSF) does not guarantee that infected people are free of the virus, its adverse immunological effects, or risk for HAND [68]. Persistent and progressive neuronal loss occurs in people with chronic HIV despite successful viral suppression by cART [69], suggesting that they are developing a concurrent neurodegenerative disorder in the setting of stable HIV infection, that HIV is causing neurodegenerative changes or that both are occurring.

### Beta-amyloid

Abnormal beta-amyloid (Aβ) accumulation is a hallmark of AD that has been found to occur in HIV [70,71]. Aβ abnormalities are more consistent in AD than in HIV, particularly among younger HIV-infected people. Increasing age is a risk factor for Aβ deposition in HIV, but recent evidence suggests that HIV and aging independently affect Aβ deposition [72], whereas AD and aging clearly interact. In HIV, plaques tend to be diffuse, and amyloid depositions commonly occur in neuronal somas as well as in extracellular plaques and along axonal tracks [70,71,73]. In AD, however, plaques are neuritic and occur particularly in extracellular space [74]. Neuropathology studies suggest that, in HIV, Aβ aggregates preferentially in the hippocampus, frontal lobe, and basal ganglia [70,75]. The location of Aβ accumulation varies in AD but tends to occur in neocortical areas first [74]. There is some evidence that long-term cART use may contribute to Aβ accumulation [70].

Aggregated Aβ can also occur in older people without cognitive disturbances, but whereas it is ubiquitous and extensive in AD [76], it is not a fundamental aspect of normal cognitive aging. Aβ aggregates in similar brain areas in healthy aging as in AD but usually more slowly and with less neurotoxicity [77]. Overall, although Aβ is highly associated with AD, evidence is limited to suggest that it is a driving force in HAND.

### Phosphorylated tau

Neurofibrillary tangles composed of hyperphosphorylated tau (pTau) are another hallmark of AD that occurs in people with HIV. Unlike amyloid plaques, pTau occurs in the majority of older adults. However, elevated pTau occurs at earlier ages in people with HIV than in healthy controls [78]. Although pTau levels appear to be unrelated to HIV viral levels in the brain [79], pTau is associated with microglial activation. Tau phosphorylation in HIV may result from pro-inflammatory cytokines and viral proteins that alter amyloidosis, which precede the formation of tau tangles [80]. Higher levels are also associated with antiretroviral treatment [78]. In the context of HIV, pTau is generally found in the hippocampus and entorhinal cortex and later spreads to surrounding areas [78], which mirrors the pattern seen in normal aging and AD [81].

### Cerebrospinal fluid markers

CSF concentrations of pTau and Aβ correspond with concentrations in the brain, although for Aβ an inverse relationship exists, reflecting problems with clearance. Elevated pTau and decreased Aβ have been reported in the CSF of people with symptomatic HIV [82], mirroring the pattern found in people with AD, although this finding has been inconsistent, particularly for total tau and pTau [83]. In one study, decreased CSF Aβ, but not elevated total tau or pTau, was found in people with HAND [84]. In contrast, elevated CSF pTau was shown in patients with asymptomatic HIV compared with controls in a recent study [85], and recent findings yet to be published by our group indicate elevated CSF pTau among older HIV-infected people with HAND. Accordingly, similarities exist between HIV and AD with respect to CSF Aβ and tau [82], although greater disturbances are found in AD, particularly compared with young adults with neuroasymptomatic HIV.

### Blood–brain barrier disturbances

The permeability of the blood–brain barrier (BBB) is altered in HIV, allowing leakage of toxic substances, including infected macrophages from the blood into the brain parenchyma. HIV affects neuronal endocytosis, which alters the integrity of the microvascular endothelial cells that compose the BBB [86]. HIV-induced disruption of the tight cell junctions and upregulation of adhesion molecules facilitate BBB passage [87]. BBB dysfunction has been linked to Aβ accumulation in HIV and other diseases resulting from a failure to filter amyloid peptides [88]. HIV has been shown to increase *in vitro* intracellular Aβ accumulation in microvascular endothelial cells [89]. BBB dysfunction is associated with neurodegeneration in AD, acting as both a cause and a consequence of cerebral Aβ accumulation, and AD and HIV share several common pathophysiological mechanisms that affect BBB permeability and Aβ accumulation [88,90].

## Risk factors and pathophysiological mechanisms

### Genetic predisposition

The apolipoprotein-E ε4 allele (ApoEε4) is a well-established AD risk factor [91] that has been associated with increased amyloid accumulation, reduced brain volumes, impaired neurocognitive functioning, and accelerated systemic progression of HIV [92,93]. ApoEε4 has been shown to increase cell susceptibility to HIV infection *in vitro* [93]. ApoEε4 has also been linked to reduced cognitive performance in HIV compared with age-matched seronegative ApoEε4^+^ participants [92], although some research does not support a significant association between ApoEε4 and HAND [94]. The relationship between ApoEε4 and cognitive functioning is more robust in AD than in HIV, as carriers with two alleles have up to a 90% chance of having AD by age 80, and ApoEε4 has been said to account for the majority of the risk associated with developing AD [91]. Although pre-existing genetic factors may influence the impact of HIV on neurological structure and function, HIV also causes epigenetic changes that may contribute to neurodegeneration and cognitive impairment as well [95].

### Cerebral metabolism

Converging lines of evidence indicate that cerebral metabolite disturbances are common among HIV-infected individuals and contribute to neurocognitive and brain abnormalities [47,87,96]. Mitochondrial disturbances in these individuals cause oxidative stress through the overproduction of reactive oxygen species (ROS), which affects viral replication, inflammation, immune function, sensitivity to drug toxicities, and HAND development [87,96,97]. The oxidative stress and cell damage caused by ROS have been proposed as a major driver underlying brain aging [98] and may also contribute to HIV effects on the aging brain, along with abnormal insulin signaling [99]. Mitochondrial dysfunction has also been associated with increased neuroinflammation, glutamate overproduction, and calcium accumulation, all of which can be neurotoxic [100]. Similarly, alterations in brain mitochondrial function, glucose metabolism, and oxygen utilization have been implicated in AD [101,102]. Oxidative stress occurs at early stages of AD and may promote the formation of Aβ plaques and tau tangles [101].

### Inflammation and neuroimmunological disturbances

HIV spreads from infected monocytes to uninfected cerebral microglia and astrocytes, activating inflammatory immune responses involving the release of cytokines, chemokines, and ROS. Chronic neuroinflammation resulting from prolonged glial and astrocyte activation has been shown to lead to neuronal dysfunction and death [87,96] and has been linked to HIV-associated brain abnormalities [97].

Regional microglial activation measured by PET has been shown to correspond to executive dysfunction in HIV [103], consistent with autopsy findings showing frontal cortical accumulation of DNA oxidative damage induced by ROS in people with AIDS [104]. Increased glial activation was found in cases of neuroasymptomatic HIV, with significant increases in frontal and parietal activation among people with HAD, suggesting that excessive glial activation and neuroinflammation precede cognitive decline [105]. PET studies show that widespread microglial activation also occurs in AD and is linked to cognitive dysfunction [106]. Similar immunological responses also occur, with Aβ accumulation leading to astrocyte upregulation and inflammatory response [90]. Neurofibrillary tangles and neuronal degeneration also promote neuroinflammation.

### Neurotoxicity

HIV-associated brain dysfunction is potentiated by a cascade of excitotoxic and apoptotic processes that amplify immunologic and inflammatory responses to the virus [87,96,99]. T-cell depletion and apoptosis are affected directly by HIV gene expression and indirectly by apoptosis in uninfected cells. Among the substances that have been implicated in HIV-associated neurotoxicity are trans-activator of transcription (Tat), glycoproteins (such as gp120), and complementary proteins (such as Fas). Both Tat and gp120 impair glutamate uptake by astrocytes, causing glutamate excitotoxicity, which leads to inflammation and apoptosis. They also cause calcium accumulation, which has similar neurotoxic effects. Furthermore, Tat can induce astrocytosis and neuronal death and interacts with amyloid precursor protein to increase Aβ [107]. These viral structure and regulatory proteins also cause cerebral mitochondrial dysfunction and ROS overproduction, causing oxidative toxicity that, as previously described, contributes to BBB dysfunction and tissue damage [97,100]. Neurotoxicity has also been implicated in AD, other neurodegenerative diseases, and normal brain aging [90,108].

Neurotoxicity may also result from the antiretroviral drugs used to treat HIV [109], particularly certain nucleoside analog reverse transcriptase inhibitors. Certain antiretroviral drugs penetrate the BBB and enter the brain more easily than others, making them good candidates to treat HIV-associated brain dysfunction [110]. Yet cART-treated HIV patients show higher levels of cerebral Aβ and pTau than cART-naïve patients in recent studies [70,78]. Findings have been mixed [30,84], but overall it seems unlikely that cART is the major cause of brain dysfunction in most patients. Nonetheless, more research on cART-associated neurotoxicity is needed, especially given the chronic cART use among people aging with HIV and the large number of new drugs under development.

Neurotoxicity also occurs indirectly as a result of infection of other organ systems outside of the brain, such as gut, liver, and vascular systems. For example, HIV causes leaky gut syndrome by infecting the gut and altering the permeability of the intestinal lining, enabling bacteria and toxins to enter the blood, which causes systemic and ultimately cerebral inflammation [111]. Hepatic ceramides produced in response to HIV have also been linked to metabolic syndrome, apoptosis, and neurodegeneration [112].

### Vascular and metabolic comorbidities

Some comorbid conditions, like chronic substance abuse, contribute to HIV transmission, functional outcomes, and cognitive problems in their own right, largely independent of the direct effects of HIV [113]. Others, like hepatitis C, exacerbate the neurocognitive effects of HIV through similar mechanisms [29,42]. Vascular and metabolic comorbidities, including diabetes, metabolic syndrome, obesity, and vascular disease, are now occurring with increased prevalence as chronically HIV-infected people age [114], and there is mounting evidence that HIV contributes to their development or expression [115]. Each of these conditions can adversely affect neurocognitive functioning [116,117]. For example, abnormal glucose metabolism leads to hyperglycemia and hyperinsulinemia, which induce ROS production, tau hyperphosphorylation, amyloid oligomerization, and widespread brain microangiopathy, and can lead to reduced Aβ clearance [116]. Thus, vascular cognitive impairment may be an important component of HAND caused by contribution of HIV to the development of vascular comorbidities. Still, the unique contribution of vascular cognitive impairment to HAND may be difficult to ascertain. It should also be emphasized that vascular risk factors are highly prevalent in the elderly, and there is strong evidence that these can be associated with vascular cognitive impairment, even in the absence of discrete cerebrovascular events [118]. Epidemiological studies have long suggested that these conditions increase risk for developing AD [116,117,119], and increased vascular risk is associated with greater amyloid burden in both HIV [120] and AD [111,121]. Given the modifiable nature of vascular and metabolic risk factors, these may ultimately be important targets for treatment as a way of preventing or diminishing cognitive dysfunction in HIV.

## Premature cognitive aging, neurodegenerative disease, or both?

The findings described above illustrate multiple commonalities as well as notable differences between the cognitive disturbances and brain dysfunction that occur secondary to HIV versus AD and other neurodegenerative diseases (Table 1). The fact that many people living with chronic HIV are experiencing cognitive and neurological decline during mid-life and resembling the neurological functioning of older adults provides compelling evidence that premature cognitive aging is occurring despite cART effectively reducing HIV-associated morbidity. Yet obvious differences exist with respect to the clinical course and cognitive domains affected in HIV and AD. Progressive cognitive decline resulting in severe dementia is ubiquitous in AD. Eventually, patients with AD experience profound cognitive dysfunction that affects memory encoding and storage, language, and higher-order intellectual abilities. This is not the case with HAND, and severe dementia is currently rare among people whose HIV is well controlled. Different trajectories of cognitive decline also exist for HIV and AD, and the usual age of onset is much younger in HAND than in AD. Furthermore, the correspondence between viral pathogen, immunological disturbance, and cognitive decline that is an essential feature of HAND does not exist for AD or most other neurodegenerative disorders. These clinical considerations might lead one to conclude that HIV and AD affect the brain and cognition in very different ways.

**Table 1:** **Summary of neurocognition, neuroimaging, neuropathology, and pathophysiology of brain disturbances in HIV and Alzheimer’s disease**

	**HIV**	**Alzheimer’s disease**	**Both**
Neurocognitive manifestations	Psychomotor slowing	Primary amnestic disturbance	Memory disturbances
	Executive dysfunction	Anomia	
	Selective cognitive impairments	Global cognitive dysfunction	
Cerebral volumetric changes	Early declines in basal ganglia and frontal lobe volumes	Greater cortical atrophy and ventricular enlargement	Early white matter changes
DTI findings	Early frontal lobe changes	Early hippocampal changes	Globally decreased FA
MRS findings	Elevated Cho		Decreased NAA
			Elevated MI
Aβ	Diffuse	Neuritic	Occur in neocortical areas
	Extracellular and intracellular	Primarily extracellular	
pTau			Elevated in medial temporal lobe
CSF markers	Inconsistent Tau findings	Elevated pTau	Decreased amyloid
ApoEε4		Robust relationship with cognitive dysfunction and dementia risk	Increases risk for Aβ, cerebral atrophy, cognitive dysfunction, and disease progression
BBB			Altered function
Glucose metabolism	Increased in basal ganglia	Decreased in parieto-temporal areas, posterior cingulate cortices, and medial temporal lobes	
	Decreased in frontal lobes		
Mitochondrial function			Impaired
Neurotoxicity			Increased
Oxidative stress			Increased
Inflammation			Increased
Vascular and metabolic influences	May occur as a result of HIV		Exacerbate cognitive effects
			Increase Aβ burden

Findings common to both diseases are listed, along with findings unique to each. Aβ, beta-amyloid; ApoEε4, apolipoprotein-E ε4; BBB, blood–brain barrier; Cho, choline; CSF, cerebrospinal fluid; DTI, diffusion tensor imaging; FA, fractional anistropy; MI, myoinositol; MRS, magnetic resonance spectroscopy; NAA, N-acetylaspartate; pTau, hyperphosphorylated tau.

Although AD and HIV share some structural, functional, and metabolic brain abnormalities and neuropathology, there are important differences. Aβ accumulation occurs at much higher rates in AD than in HIV or normal aging, while pTau findings are inconclusive. Furthermore, neurodegeneration generally occurs in hippocampal regions first in AD, whereas HIV shows a fronto-subcortical pattern. AD and HIV share some common pathophysiological mechanisms, including altered BBB activity, oxidative stress, and neuroinflammation, but HIV has a number of more specific brain effects caused by toxic glycoproteins, such as gp120, ongoing viral replication in certain brain areas despite overall systemic and CSF viral suppression, HIV co-receptors on lymphocytes, macrophages, neurons and microglial cells, and genetic or epigenetic alterations in response to the virus that may cause neuronal damage. These specific pathophysiological mechanisms obviously differentiate HIV from AD. The extent to which AD is caused by related mechanisms remains to be determined.

### The verdict

Based on existing evidence, several conclusions can be reached. HIV causes premature cognitive and brain aging. These effects are caused by direct damage from the virus as well as indirectly through increased risk of cardiovascular disease, chronic drug use, and potentially toxic long-term antiretroviral use. There has been some controversy over whether HIV causes neurodegeneration as such, and over whether HAND should be considered a neurodegenerative disease. Evidence for neurodegeneration is provided by recent longitudinal studies showing declines in memory and other cognitive functions over time as well as relatively high rates of HIV-infected people transitioning from being asymptomatic to having HAND. Brain pathophysiology also suggests that HIV causes neurodegenerative changes, at least in some people. Despite the many commonalities between HIV and AD, it seems unlikely that HIV causes AD *per se*. Besides differences in their usual clinical, cognitive, and neuropathological presentation, HIV is clearly caused by virus, whereas AD is not. Nonetheless, common pathophysiological pathways exist in HIV and typical neurodegenerative diseases that contribute to accelerated age-associated cognitive decline. In the current era of cART, when HIV can be well controlled from the time of diagnosis, it may be that people will not show the same effects of chronic infection as they age, or at least not to the same degree. Future research is necessary to address this question, examining newer cohorts of HIV-infected people who have not experienced severe immunocompromise. Beyond targeting viral replication with cART, targeting vascular and metabolic factors will likely be important for delaying or mitigating HAND. Future studies should examine whether modifying these factors through clinical interventions results in improved cognitive function in people with HIV.

## Abbreviations

AD: Alzheimer’s disease
ADC: AIDS dementia complex
ANI: Asymptomatic neurocognitive impairment
ApoEε4: Apolipoprotein-E ε4
Aβ: Beta-amyloid
BBB: Blood–brain barrier
cART: Combination antiretroviral therapy
Cho: Choline
Cr: Creatine
CSF: Cerebrospinal fluid
DTI: Diffusion tensor imaging
gp120: Glycoprotein 120
HAD: HIV-associated dementia
HAND: HIV-associated neurocognitive disorder
MI: Myoinositol
MND: Mild neurocognitive disorder
MRI: Magnetic resonance imaging
MRS: Magnetic resonance spectroscopy
NAA: N-acetylaspartate
PET: Positron emission tomography
pTau: Hyperphosphorylated tau
ROS: Reactive oxygen species
Tat: Trans-activator of transcription
WMH: White matter hyperintensity
